# Medication Adherence Among Diabetic and Hypertensive Patients in Rural Tamil Nadu: A Cross-Sectional Study

**DOI:** 10.7759/cureus.90998

**Published:** 2025-08-25

**Authors:** Shankar GK, Kannan L

**Affiliations:** 1 Community Medicine, Sri Ramachandra Institute of Higher Education and Research, Chennai, IND

**Keywords:** diabetes, hypertension, medication adherence, monotherapy, non-communicable disease

## Abstract

Introduction

Diabetes and hypertension are prevalent non-communicable diseases in India, with poor medication adherence contributing to complications, hospitalizations, and increased healthcare burden. This research was done to assess the adherence to medication and factors associated with it among patients with diabetes and hypertension in rural Tamil Nadu.

Materials and methods

A hospital-based analytical cross-sectional study was conducted over three months (April-June 2025) among diabetic and hypertensive patients attending the outpatient department of a Rural Health Centre in Tiruvallur, Tamil Nadu. Adults diagnosed with diabetes mellitus and/or hypertension for at least one year and who consented to the study were included. The required sample size was 174, based on a prevalence of 67.1% medication adherence, and 195 participants were ultimately enrolled. Data were collected using a pretested questionnaire covering sociodemographic details, disease status, and the Simplified Medication Adherence Questionnaire. Ethical clearance was obtained from the Institutional Ethics Committee of Sri Ramachandra Institute of Higher Education and Research (approval number: CSP-MED/25/FEB/113/30). Statistical analysis was done using SPSS Statistics (IBM Corp., IBM SPSS Statistics for Windows. Armonk, NY: IBM Corp.).

Results

The age of study participants ranged from 35 to 85 years, and the average age was 57.05 years. Most of the participants were females (53.3%, n=104); 36.9% (n=72) of the participants had only diabetes, 24.6% (n=48) had only hypertension, and 38.5% (n=75) had both conditions. The prevalence of medication adherence was 58.5% (n=114, 95% CI: 51.4-65.2). Patients aged ≤50 years (OR: 2.19, p=0.020) and those on monotherapy (OR: 2.03, p=0.033) were significantly more adherent. Other variables such as sex, marital status, socioeconomic status, and comorbidities were not significantly associated with adherence.

Conclusions

Nearly half the patients were non-adherent, posing a public health concern. Age and complexity of drug regimen were significant predictors of adherence. Interventions such as patient education, fixed-dose combinations, and digital reminders should be prioritized to improve adherence and reduce complications.

## Introduction

Diabetes mellitus (DM) and hypertension (HTN) are two of the most prevalent non-communicable diseases worldwide, contributing significantly to the global burden of chronic illnesses and posing significant public health challenges. Their prevalence continues to increase because of rapid urbanization, poor lifestyle, and unhealthy diets. The burden is particularly high in India, with a growing population affected by these conditions, posing a significant issue affecting public health.

In India, there is a rapid increase in the number of people with DM and HTN. According to a Metabolic Non-communicable Disease Health Report of India by ICMR-INDIAB 17 published in 2023, DM had a prevalence of 11.4%, and the overall prevalence of HTN was 35.5% [1].

The same study found that in Tamil Nadu, the total prevalence of DM was more than 10% and the prevalence of HTN was more than 30%. The TN STEPS survey conducted in 2020 showed the prevalence of DM to be 17.6% and the prevalence of HTN to be 33.9% [2].

The high burden of DM and HTN significantly contributes to increased morbidity, mortality, and healthcare costs. Poor blood pressure and glycemic control often lead to complications such as cardiovascular diseases, kidney failure, and stroke [3]. A key factor contributing to suboptimal disease control is inadequate medication adherence. Non-adherence reduces treatment efficacy, worsening disease progression, and increasing hospitalizations. This places a significant strain on healthcare systems and economies. Improving adherence is critical to reducing the health and economic burden of these chronic diseases. Therefore, this study aims to evaluate the adherence to medication among patients suffering from DM and HTN.

Objectives

This study aims to assess adherence to medication among patients with DM and HTN. To identify factors influencing adherence and to compare the adherence between patients on monotherapy and combination therapy.

## Materials and methods

Study design and setting

This cross-sectional study was conducted within a hospital setting for a duration of three months, from April to June 2025. The study population included diabetic and hypertensive patients visiting the outpatient department of the Rural Health and Training Centre, which is associated with a medical college and located in Tiruvallur district, Tamil Nadu.

Inclusion and Exclusion Criteria

Participants included in the study were adults diagnosed with DM or HTN, or both, for a minimum duration of one year, and who consented to participate in the study. Patients diagnosed with DM or HTN for less than one year, those with severe acute illness requiring immediate hospitalization, individuals with impaired decision-making ability or psychiatric conditions that could interfere with informed consent or study participation, and those unwilling to provide consent were excluded from the study.

Sample size

The study sample size was calculated based on a medication adherence prevalence of 67.1%, as reported in a previous study conducted by Mishra et al. among patients with DM attending the non-communicable disease clinic [4]. With a 95% confidence level, 90% power (β error of 10%), and precision of 7%, the calculated sample size was 174. All the patients attending the outpatient department who satisfied the inclusion criteria were taken up for the study. The final sample size was 195.

Data collection

A brief description of the study was given to the participants before starting the collection of data. The questionnaire was administered in the outpatient room with adequate privacy for the participant. Written consent was collected from every participant after informing them about the study. Part 1 of the questionnaire consisted of sociodemographic characteristics of the study participants. The most recent blood pressure, fasting, and postprandial blood sugar values in the patient’s record were noted. Part 2 of the questionnaire consisted of details about the non-communicable disease status of the participants. The Simplified Medication Adherence Questionnaire adapted for diabetes and hypertension was used to interview the participants [5]. The study was carried out after receiving approval from the Institutional Ethics Committee of Sri Ramachandra Institute of Higher Education and Research (approval number: CSP-MED/25/FEB/113/30).

Data analysis

The data collected was recorded into Microsoft Excel (Microsoft Corp., Redmond, WA, USA), and the statistical analysis was carried out using SPSS Statistics (IBM Corp., IBM SPSS Statistics for Windows. Armonk, NY: IBM Corp.) [6]. Descriptive analysis was performed to summarize the sociodemographic characteristics, health status, and medication adherence of the study participants. Categorical variables were presented as frequencies and percentages. Univariate analysis using the chi-square test of significance was done to assess the association between medication adherence and relevant independent variables. A p-value of less than 0.05 was considered statistically significant.

## Results

One hundred ninety-five diabetic patients attending the Rural Health and Training Centre were included in this study. The majority of the patients were females (53.3%). The age of the patients varied from 35 years to 85 years, and the average age was 57.05 years. 70.3% of the study participants were literate. The sociodemographic details of the study participants are mentioned in Table 1.

**Table 1:** Sociodemographic characteristics of study participants determined using the modified BG Prasad Scale 2025

S. No.	Characteristic	n (%)
1	Age distribution	
	≤50 years	56 (28.7%)
	>50 years	139 (71.3%)
2	Sex	
	Male	91 (46.7%)
	Female	104 (53.3%)
3	Level of education	
	Illiterate	58 (29.7%)
	Primary	36 (18.5%)
	Secondary	64 (32.8%)
	Higher secondary	21 (10.8%)
	Graduate	16 (8.2%)
4	Occupation	
	Unemployed	46 (23.6%)
	Unskilled	60 (30.8%)
	Semi-skilled	54 (27.7%)
	Skilled	35 (17.9%)
5	Marital status	
	Married	149 (76.4%)
	Unmarried	46 (23.6%)
6	Number of family members	
	≤2	79 (40.5%)
	3-4	69 (35.4%)
	≥5	47 (24.1%)
7	Socioeconomic status (modified BG Prasad Scale 2025)	
	Upper	11 (5.6%)
	Upper middle	55 (28.2%)
	Middle	67 (34.4%)
	Lower middle	40 (20.5%)
	Lower	22 (11.3%)

Among the participants, the largest group, 38.5% (75 individuals), had both DM and HTN; 36.9% (72 individuals) had only DM, and 24.6% (48 individuals) had only HTN. The majority of participants, 70.8% (138 individuals), were receiving combination therapy for their conditions. Among diabetics, 74.8% (110 individuals) monitored their condition at least every three months. Among hypertensives, 82.9% (102 individuals) reported monitoring their blood pressure every two weeks or more often. A little over half of the study participants (54.9%) had a positive family history of DM or HTN. Among those with a history of hospitalization, the leading cause was uncontrolled DM, accounting for 14.9% (29 individuals). Uncontrolled HTN led to hospitalization for 6.7% (13 individuals), while 3.1% (six individuals) were hospitalized due to both uncontrolled DM and HTN. Only half the participants (n=98, 50.3%) had good blood pressure and glycemic control during the study; 25.6% (50 individuals) had a history of complications related to DM and HTN, which can be seen in Table 2.

**Table 2:** Health status of study participants Monotherapy: 1 drug, combination therapy: >1 drug IHD: ischemic heart disease, CKD: chronic kidney disease

S. No.	Characteristic	n (%)
1	Disease condition	
	Diabetes	72 (36.9%)
	Hypertension	48 (24.6%)
	Both	75 (38.5%)
2	Number of drugs taken	
	Monotherapy	57 (29.2%)
	Combination therapy	138 (70.8%)
3	Frequency of monitoring	
	Diabetes	
	≤3 months	110 (74.8%)
	>3 months	37 (25.2%)
	Hypertension	
	≤2 weeks	102 (82.9%)
	>2 weeks	21 (17.1%)
4	Family history of diabetes/hypertension	
	Present	107 (54.9%)
	Absent	88 (45.1%)
5	Other comorbid conditions	
	Present	32 (16.4%)
	Absent	163 (83.6%)
6	Type of comorbid condition	
	Hyperlipidemia	21 (65.6%)
	Hypothyroidism	6 (18.8%)
	Bronchial asthma	5 (15.6%)
7	History of hospitalization	
	Uncontrolled diabetes	29 (14.9%)
	Uncontrolled hypertension	13 (6.7%)
	Both	6 (3.1%)
	No	147 (75.3%)
8	Blood pressure and glycemic control	
	Yes	98 (50.3%)
	No	97 (49.7%)
9	Complications	
	Diabetic foot	14 (7.2%)
	Peripheral neuropathy	11 (5.6%)
	IHD	9 (4.6%)
	CKD	6 (3.1%)
	Stroke	7 (3.6%)
	Carbuncle	3 (1.5%)
	No	145 (74.4%)

The participants were asked about their medication adherence status using the following questions from the Simplified Medication Adherence Questionnaire adapted for DM and HTN. The prevalence of medication adherence among the study participants was 58.5% (n=114, 95% CI: 51.4 - 65.2). The details of the answers given by the participants are summarized in Table 3.

**Table 3:** Details of medication adherence among study participants The Simplified Medication Adherence Questionnaire, adapted for diabetes and hypertension, was used to interview the participants [5].

S. No.	Characteristic	n (%)
1	Do you ever forget to take your medication for diabetes and/or hypertension?	
	Yes	57 (29.2%)
	No	138 (70.8%)
2	Do you always take your medication for diabetes and/or hypertension at the indicated time?	
	Yes	174 (89.2%)
	No	21 (10.8%)
3	Sometimes, if you feel worse, do you stop taking your medication for diabetes and/or hypertension?	
	Yes	24 (12.3%)
	No	171 (87.7%)
4	Did you miss your medication for diabetes and/or hypertension last weekend?	
	Yes	40 (20.5%)
	No	155 (79.5%)
5	How often did you miss your medication for diabetes and/or hypertension last week?	
	Never	123 (63.1%)
	1-2 doses	27 (13.8%)
	3 -5 doses	26 (13.3%)
	6-10 doses	11 (5.6%)
	>10 doses	8 (4.1%)
6	Have you missed your medication for diabetes and/or hypertension in the past three months?	
	Yes	78 (40%)
	No	117 (60%)
7	Medication adherence	
	Adherent	114 (41.5%)
	Non-adherent	81 (58.5%)

Patients less than or equal to 50 years and patients receiving monotherapy were seen to be associated with medication adherence, with statistically significant odds ratios of 2.19 and 2.03, respectively. The other variables, like sex, marital status, socioeconomic status, family history, and presence of other comorbid conditions, had no association with medication adherence. The details of the OR, 95% CI, and p-value are given in Table 4 for all the variables.

**Table 4:** Univariate analysis of factors associated with medication adherence (Chi-square test)

S. No.	Characteristic	Medication adherence		Odds ratio (95% CI)	p-value
		Yes	No		
1	Age				
	≤50 years	40	16	2.19 (1.12-4.28)	0.020
	>50 years	74	65		
2	Sex				
	Female	64	40	1.31 (0.74-2.32)	0.351
	Male	50	41		
3	Marital status				
	Married	87	62	0.98 (0.50-1.93)	0.971
	Unmarried	27	19		
4	Socioeconomic status				
	Upper	42	24	1.38 (0.75-2.55)	0.294
	Lower	72	57		
5	Family history				
	Yes	62	45	0.95 (0.53-1.69)	0.872
	No	52	36		
6	Number of drugs				
	Monotherapy	40	17	2.03 (1.05-3.93)	0.033
	Combination	74	64		
7	Other comorbid conditions				
	Present	18	14	0.89 (0.41-1.92)	0.781
	Absent	96	67		

## Discussion

In this study, the prevalence of medication adherence was 58.5% (95% CI: 51.4-65.2). This is similar to a study done in Pondicherry, which found good medication adherence of 60.5% among its study participants [7]. However, it is higher than another study in Thiruvarur, Tamil Nadu, which found only 42.4% good adherence among NCD patients [8]. It is also higher than the 38.61% medication adherence reported for hypertensive patients in Ooty, South India [9]. However, the adherence rate of this study is lower than results from a study in Kancheepuram, Tamil Nadu, which found 84% overall adherence to medication in patients with HTN and DM, and a study in Villupuram, Tamil Nadu, where 85.7% of NCD patients demonstrated good adherence [10,11].

In other regions of India, our findings were consistent with those of a study conducted in Rohtak, North India, which reported a medication adherence rate of 58.6% among patients with HTN [12]. A higher adherence rate was seen in some studies, such as those in Chandigarh (79.5% for diabetic patients), Kolkata, West Bengal (69.7% high adherence among diabetic patients), and Odisha, Eastern India (67.1% for diabetic patients) [4,13,14]. It is also significantly lower than the very high adherence of 91.3% reported for hypertensive patients in Warangal, Telangana [15]. Another study done in Eastern India reported good medication adherence in only 34.14% of diabetic patients [16]. In Davangere, Karnataka, 45% of diabetic patients were highly adherent, and in Uttarakhand, 44% showed good adherence among diabetic patients [17,18]. The variation in medication adherence across different regions may be due to differences in health literacy, accessibility to healthcare services, and medication costs. Factors such as cultural beliefs, socioeconomic status, and the presence of effective health education or community support programs may also influence adherence levels.

Findings from Table 4 show that age as well as the type of drug therapy were seen to be associated with medication adherence among the participants. Individuals aged 50 years or below had over two times higher odds of adhering to medication compared to those above 50 years (OR: 2.19, 95% CI: 1.12-4.28, p=0.020). This may be due to younger individuals being more active, literate, health-conscious, and generally more responsive to health advice and treatment plans, which can lead to better adherence. Similarly, those on monotherapy were significantly more likely to adhere to treatment than those on combination therapy (OR: 2.03, 95% CI: 1.05-3.93, p=0.033). This shows that simpler medication regimens may be easier for patients to adhere to. This may also be due to the reduced pill burden and fewer side effects.

Other variables such as sex, marital status, socioeconomic status, family history of illness, and presence of other comorbidities did not show any statistically significant association with medication adherence in this study (p>0.05). A study in Puducherry found that females had poor medication adherence compared to males (p=0.001) [7]. Similarly, a study in a rural population of Kerala reported that females had a higher risk of poor adherence than males (OR: 1.854, 95% CI: 1.039-3.308, p=0.036) [19]. A study in Gujarat on hypertension found that non-adherence was lower among married (19.2%) participants than among unmarried participants (64.4%), and this relationship was statistically significant. The same study found higher non-adherence among participants with a family history of hypertension, and this relationship was statistically significant (p=0.0001) [20]. In Bhopal, Madhya Pradesh, participants of lower socioeconomic classes were seen to have a low adherence, and this relationship was statistically significant (p=0.0001) [21]. The lack of statistical significance for these variables in our study may be due to variations in population characteristics.

While the study provides valuable insights, certain limitations may be noted. As it was cross-sectional in design, causal relationships between factors and medication adherence could not be established. The use of a questionnaire-based assessment may be affected by a recall bias, especially among older participants. However, this appears to be among the first studies to assess medication adherence among both diabetic and hypertensive patients using the validated Simplified Medication Adherence Questionnaire [22], and the results were comparable to those of existing studies that employed different assessment scales. The inclusion of patients with both conditions adds to the comprehensiveness of the findings and enhances their applicability to non-communicable disease management programs.

## Conclusions

The findings obtained from this study indicated that almost half of the participants were nonadherent to diabetic and hypertensive medications, which is quite a large number, indicating a significant public health concern. Various factors have been identified to be associated with medication nonadherence by studies in different regions. There is an urgent need to focus on the obstacles or barriers to medication adherence. This study suggests providing targeted intervention for patients aged more than 50 years and patients on combination therapy. Healthcare providers should spend more time providing health education regarding the adverse outcomes of medication nonadherence and the complications arising as a result of uncontrolled DM and HTN. Information, education, and communication materials can be displayed in hospitals and other areas commonly used by the general public. Strategies like mobile app reminders and digital follow-up systems should be considered in routine non-communicable disease management. Prescribing multiple individual medicines for the same disease can overwhelm patients and lead to poor adherence. Adopting fixed-dose combination therapies may simplify treatment and improve compliance. Improving medication adherence will result in a better prognosis, fewer complications, and a decrease in overall disease burden on the healthcare system.
